# High burden of neural tube defects in Tigray, Northern Ethiopia: Hospital-based study

**DOI:** 10.1371/journal.pone.0206212

**Published:** 2018-11-14

**Authors:** Birhane Alem Berihu, Abadi Leul Welderufael, Yibrah Berhe, Tony Magana, Afework Mulugeta, Selemawit Asfaw, kibrom Gebreselassie

**Affiliations:** 1 Department of Anatomy, Institute of Biomedical Sciences, College of Health Sciences, Mekelle University, Mekelle, Ethiopia; 2 Department of pediatrics and child health, School of medicine, College of Health Sciences, Mekelle University, Mekelle, Ethiopia; 3 Department of Obstetrics and Gynecology, School of medicine, College of Health Sciences, Mekelle University, Mekelle, Ethiopia; 4 Department of Surgery, School of medicine, College of Health Sciences, Mekelle University, Mekelle, Ethiopia; 5 Department of nutrition, School of Public Health, College of Health Sciences, Mekelle University, Mekelle, Ethiopia; University of Ottawa Faculty of Medicine, CANADA

## Abstract

**Introduction:**

Neural tube defects are the major causes of fetal loss and considerable disabilities in infants. Currently, there is no significant research on the incidence of Neural tube defects in the Tigray region of Ethiopia.

**Objective:**

To determine the incidence and clinical pattern of the Neural Tube Defects.

**Methods:**

A hospital-based cross-sectional study was conducted from October 2016 to June 2017. All pregnancy outcomes were examined for any externally visible birth defects and neurological integrity by trained midwives under the supervision of senior obstetrics and gynecology and a neurosurgeon. Data were collected using a survey tool to collect maternal and newborn demographic data and a checklist developed to capture newborns with Neural Tube Defects. Data were analyzed using SPSS version 20. The prevalence of NTDs was calculated per 10,000 births.

**Result:**

Out of the 14,903 births during the study period, a total of 195 infants were born afflicted with Neural Tube Defects. The burden of infants with anencephaly and spina bifida was 66.4 and 64.4 per 10, 000 births, respectively. The overall incidence rate of NTDs in this study was 131 per 10, 000 births of which 23% were liveborn and 77% were stillborn. The highest burden of Neural Tube Defects was observed in Adigrat Hospital from Eastern Zone of Tigray (174 per 10,000 births) and Lemlem Karl Hospital from Southern Zone of Tigray (304 per 10,000 births) compared to Kahsay Abera Hospital from Western Zone (72.8 per 10,000 births) and Sihul Hospital from North Western Zone of Tigray (69.8 per 10,000 births).

**Conclusion and recommendation:**

Assuming that the non folic acid preventable rate should be 5 per 10,000 births, our prevalence rate is 131 per 10,000 births, and then we have a rate or an epidemic that is 26 times what it should be. This just emphasizes the urgency to implement effective programs to get all women of reproductive age to have adequate folic acid to prevent all of folic acid-preventable spina bifida and anencephaly, which would prevent 96% (125/130) of spina bifida and anencephaly in the Tigray Provence.

## Introduction

Neural tube defects (NTDs) are the developmental malformation of the central nervous system. Worldwide, about 300,000 babies are born affected with NTDs each year, resulting in around 88,000 deaths and 8.6 million lifelong disabilities [1]. Worldwide, more than 10% of neonatal mortality is caused by embryological malformation of the nervous system [2]. As morbidity and mortality from infectious diseases are decreasing globally, the contribution of congenital anomalies to under five morbidities and mortality will continue to increase proportionally [1]. Though the prevalence of NTDs has reduced recently in the developed world [3,4], it still remains high in the developing world with an estimated prevalence rate ranging between 1 to 11/1000 births [5, 6]. The commonest NTD cases are Anencephaly and Spina bifida, which typically occur due to losing the integrity of the brain and spinal cord tissues. Anencephaly is a fatal NTD, but babies with Spina bifida often survive following surgical intervention [7]. Multifarious factors are involved in the development of NTDs. These defects are thought to result in part from genetic susceptibility and environmental factors such as nutritional deficiency, poverty, obesity, maternal health, and medicinal drug used [8, 9].

Various clinical and experimental studies have been reported that mandatory folic acid fortification and periconceptional supplementation can prevent the occurrence and recurrence of NTDs [10, 11, 12]. Comparably, studies showed that the risk of NTDs among all US women dropped considerably from a pre-fortification (1988–94) estimated 35.9 (95% uncertainty interval 28.1 to 46.2) NTDs per 10 000 births to a post-fortification (2005–10) 14.6 (12.4 to17) NTDs per 10 000 births [13, 14]. Similarly, a study conducted in China revealed that when there is an adequate folic acid in the mother's blood at the time of conception, the expected achievable prevention of spina bifida and anencephaly is approximately 5 to 6 per 10,000 births [15]. This suggests that folate deficiency in pregnant women has a potential for impact on the prevalence of NTDs. Information related to NTDs is limited in Tigray and hence the true incidence rate of NTDs in Tigray is unknown. Thus, the objective of this study was to determine the incidence and clinical pattern of neural tube defects in the Tigray region, Northern Ethiopia.

## Materials and methods

### Study area, period and design

The study was conducted in the Tigray region, which is one of the nine regional states of Ethiopia. Tigray region has a total population of 5,056000, with urban residency for 20% of the total population. In the region, there are 992, 635 households, with urban households having on average 3.4 and rural households 4.6 people [16]. The standard of living in Tigray region is distributed as 31.6% of the inhabitants fall into the lowest wealth quintile; adult literacy for men is 67.5% and for women 33.7% [17]. The Ethiopian Demographic and Health Survey (EDHS) 2016 showed that the coverage of ANC1 (antenatal care of at least one visit) and ANC 4+ (at least four visits) was 90% and 57%, respectively. Similarly, the EDHS 2016 reported that about 56.9% of the births in the Tigray region occur in health facilities [18].

The facility based cross-sectional study was conducted in eight randomly selected major public hospitals from the Tigray region of Ethiopia, namely Mekelle and Ayder hospitals from Mekelle Zone; Lemelem Karl hospital from Southern Zone; St. Mary hospital from Central Zone; Sihul hospital from Northwestern zone; Adigrat and Wukro hospitals from Eastern zone and Kahsay Abera Hospital from Western Zone. These hospitals serve populations with diverse demographic and economic and health characteristics. They provide 24-hour obstetrics and gynecology care for their representative population. This study was conducted from October 2016 to June 2017.

### Study population

All pregnant women who delivered in the public hospitals during the study period and consented to participate in the study were included. A prospective registration of all birth outcomes of any gestational age was conducted during the study period to determine the burden of NTDs in the region. All newborns delivered during the study period and a permanent resident of the study setting (women who lived in the representative area for the 6 months prior to delivery) were included in this study. Women outside the usual catchment area of the hospitals and the birth outcomes that are not stillbirths or live births were excluded from the study. Cases were identified at delivery rooms soon after birth and were defined as live births or stillbirths with NTDs. The stillbirths included in this survey were from spontaneous abortion, but not from induced abortions due to poor prenatal diagnosis of congenital anomalies in the governmental hospitals.

### Sample size and techniques

The minimum sample size required was 14903 based on an NTD prevalence estimate of 0.6% extrapolated from the report of the two Teaching Hospitals in Addis Ababa [16], with a confidence interval of 95% and error set at 5%. The sample size was calculated using Epi Info version 3.03a, 2015.

### Data collection tools

The NTDs survey tool maternal and newborn demographic for socio-demographic, socioeconomic, maternal health and nutrition characteristics and a template/checklist to record the result of the physical, clinical and neurological examinations of newborns with NTDs were developed by the study team. The survey tool included collecting the patient registration number to avoid duplication of cases, along with the name of the delivery hospital, date of birth, sex of the newborn, and type of neural tube defect. All birth outcomes were examined for any externally visible birth defects. The same physical examinations were done to fetuses or embryos born before term as well as the stillbirths.

### Clinical examination

All newborn babies (live and stillbirths) were carefully examined for external birth defects. Their maturity scale, location, and characteristics of the defect were examined. Neurological examinations such as motor examination of the extremities: voluntary motor movement 0 to 5 (0 = none; 1 = trace; 2 = not against gravity; 3 = gravity; 4 = some resistance; 5 = maximal resistance), tone spastic or flaccidity present and presence of neonatal reflexes or primitive reflexes (Moro reflex, tonic neck reflex, grasping reflex, sucking reflex, blink reflex) were examined, measured and recorded in the NTDs template.

### Data collection techniques and quality assurance

Data were abstracted daily by trained midwives who were trained on the pathophysiology of NTDs and comprehensive assessment of newborns with NTDs before the start of the study. Following the theoretical training, the midwives were attached to a hospital of similar setting to the study hospitals to practice the assessment and classification of NTDs and their interviewing techniques. The NTD survey tool was pre-tested in the general pediatric ward, pediatric critical care unit and delivery ward of the Ayder Comprehensive Specialized Hospital, which is the main teaching hospital of Mekelle University, College of Health Sciences and the tertiary referral hospital, for the Tigray region as well as Northern Ethiopia. Each participant was given the chance to evaluate a normal newborn and one newborn with NTD. Based on the outputs from the pretest, the survey tool was revised accordingly. The trained midwives critically follow whenever suspected cases of NTDs were delivered or medically terminated. Subsequently, data were collected at a postpartum or postabortal period and before the discharge of the women. NTDs were defined as cases of anencephaly and spina bifida among birth outcomes of any gestational age infants afflicted with NTDs. Cases were confirmed by senior obstetrics and gynecology working in the representative hospitals of Tigray region through physical examination. Any defect(s) identified were described and recorded. For the record and reference purposes, medical record numbers of the NTDs cases were taken. Mothers were counseled and advised accordingly on defects that warrant urgent medical/surgical attention. A referral system was arranged to cater for babies whose defects need professional care elsewhere. All these clinical investigations were done by the trained research assistant under the supervision of gynecologists and a neurosurgeon.

### Statistical data analysis

The data collected was coded; cleaned and analyzed using Statistical Packages for Social Sciences (SPSS version 20). Simple descriptive statistics were used to characterize the study subjects. The prevalence of NTDs was calculated per 10,000 births. The results were presented in the forms of tables and graphs.

### Ethical considerations

The study was granted an ethical approval from the Institutional Review Board of the College of Health Sciences at Mekelle University (ERC 0837/2016). For purposes of obtaining an informed consent of the respondents, every mother, on an individual basis, was furnished with detailed information concerning the research objectives, benefits and the importance of participation of the mothers and their newborn babies. It was explained that the choice to participate in the research was completely voluntary even after giving consent to participate, shall retain the right to opt out of the research, any time they feel like without repercussion against them. All information obtained was strictly kept confidential and were only being used for purposes of the research. A respondent who consents to participate was confirmed by appending her signature or thumbprint on the availed consent form.

## Result

### The incidence of neural tube defects (NTDs)

Out of the 14,903 births (live and stillbirths) during the study period, a total of 195 (126 males and 69 females) infants had NTDs. The incidence of infants with anencephaly and spina bifida was 66.4 and 64.4 per 10,000 births, respectively. The overall occurrence of NTDs was 131 per 10, 000 births (95% CI, 113–150.6). The occurrence of live and stillbirths with NTDs was 30.2 per 10, 000 births and 100.6 per 10, 000 births respectively. When the data are further disaggregated by zones, 166.6, 304, 89, 132, 72.8, 69.8 per 10, 000 births were observed in Mekelle, Southern, Central, Eastern, Western and Northwest zones of Tigray, respectively (Table 1).

**Table 1:** Facility-based incidence of neural tube defects (NTDs) in Tigray, Northern Ethiopia (n = 14,903).

Hospital	Total Deliveries registered	Total NTDs identified	NTDs per 10,000 births	NTDs per 10,000 births						
				95% Poisson		NTD type		Live Births	Stillbirths	
				LL	UL	Ane	SB	SB	Ane	SB
Ayder	2401	40	166.6	119.0	226.9	87.5	79.1	79.1	87.5	0.0
Lemelem	889	27	304	200.1	441.9	202.5	101.2	45.0	202.5	56.2
Wukro	1358	18	132.5	78.6	209.5	81.0	51.5	44.2	81.0	7.4
Kahsay	961	7	72.8	29.3	150.1	10.4	62.4	31.2	10.4	31.2
St. Mary	1685	15	89.0	49.8	146.8	41.5	47.5	0.0	41.5	47.5
Adigrat	2075	36	174	121.5	240.2	81.9	91.6	24.1	81.9	67.5
Mekelle	3529	38	107.7	76.2	147.8	45.3	62.3	11.3	45.3	51.0
Sihul	2005	14	69.8	38.2	117.2	39.9	29.9	20.0	39.9	10.0
Total	14903	195	131	113.1	150.6	66.4	64.4	30.2	66.4	34.2

NTDs: Neural tube defects; LL: Lower limit; UL: upper limit; Ane: Anencephaly; Sb: Spina bifida.

High burden of NTDs was observed in males, 65% (126/195). Specifically, Anencephaly was commonly observed in males accounting for 31% (60/195) and 20% (39/195) in females, respectively. Spina bifida was also observed in 34% (66/195) and 15% (30/195) of males and females, respectively. Among the total NTD cases, only 23% (45/195) of the infants were born alive and 77% (150/195) were stillbirths (Table 2).

**Table 2:** Characteristics of the observed neural tube defects at the time of delivery (n = 195).

**Characteristics**	**Types of neural tube defects n (%)**		**Total: n (%)**
	Anencephaly: **n (%)**	Spina bifida: **n (%)**	
Male	60(31)	66(34)	126(65)
Female	39 (20)	30(15)	69(35)
Total	99(51)	96(49)	195(100)
**Pregnancy outcome at the time of delivery n (%)**			
	Anencephaly **n (%)**	Spina bifida **n (%)**	**Total: n (%)**
Stillbirth	99(51)	51(26)	150(77)
Live birth	-	45(23)	45(23)
Total	99(51)	96(49)	195 (100)

Anatomical location of the spina bifida cases was cervical 29% (28/96), thoracic 28% (27/96) and lumbar 43% (41/96) regions, respectively. Craniorachischisis which is a rare type NTDs was also observed in one patient. The salient characteristic of the observed Spina bifida was Purulent followed by Cystic, Membranous, and Epithelialized, respectively. The most associated anomalies observed in this study were hydrocephalus and clubfoot identified in 8.7% (17/195) and 13.3% (26/195), respectively. Abdominal anomaly (omphalocele) in 2.6% (5/195) was also observed associated with spina bifida (Table 3).

**Table 3:** Anatomical location of neural tube defects and their associated anomalies.

Anatomical region		Spina bifida as per anatomical location (n = 96)	
		Frequency	Percentage (%)
Cervical		28	29
Thoracic		27	28
Lumbar		41	43
Total		96	100
**Salient characteristics of the observed Spina bifida (n = 96)**			
Spina bifida (n = 96)		Cystic	33	34.4
		Epithelialized	4	4.2
		Membranous	16	16.7
		Purulent	43	44.7
Total			96	100
**Associated congenital anomalies with neural tube defects (n = 195)**			
Yes	Abdominal anomalies	5	2.6
	Clubfoot	17	8.7
	hydrocephalus	26	13.3
No		147	75.4
Total		195	100

General neurological examination of live babies afflicted with NTDs has shown that the majority of the live infants with NTDs showed distorted neonatal reflexes such as mental status, sucking, grasping, sound, Moro, blinking and tonic neck (Table 4). The most common problems of the motor function identified in the present study were losing (power grade 0) of the upper and lower extremities (Table 5).

**Table 4:** Neonatal reflexes of live birth complicated with NTDs (n = 45).

Reflexes	Stimulation	Normal response	Frequency N (%)		
			Present	impaired	Absent
Mental status	Physical contact	Arouses with physical stimulation	14(31)	12(27)	19(42)
Sucking	Mouth touched by an object	Suck on object	10(22)	29(64)	6(13)
Grasping	Palm touching	Closes eyes	14(31)	31(69)	
Sound	Sudden Sound	Move in the sound direction	6(13)	39(87)	
Moro	Sudden move	Throws out arms and legs and then pull towards the body	2(4.4)	21(47)	22(49)
Blinking	Flash of light	Close eyes	40(89)		5(11)
Tonic neck	Placed on back	Makes fists and turns head to the right	6(13)	3(6.5)	36(80)

**Table 5:** Voluntary movement (motor) examination of live births complicated with NTDs (n = 45).

Motor examination	Power Grade N (%)					
	Grade– 0	Grade- 1	Grade -2	Grade -3	Grade -4	Grade -5
Right and left Upper extremity flexion and extension movements	17(38)	1	2(4.4)	12(27)	6(13)	7(16)
Right and left Hip flexion and extension movements	22(49)	2(4.4)	2(4.4)	13(29)	5(11)	1
Right and left Knee flexion and extension movements	24(53)	3(7)	2(4.4)	11(24)	4(9)	1
Right and left Ankle dorsiflexion or both	27(60)	1	2(4.4)	8(18)	6(13)	1
Right and left Ankle plantar flexion	25(56)	4(9)	2(4.4)	7(16)	6(13)	1

Power grade: x/5: 0 = flaccid, 1 = trace, 2 = not against gravity, 3 = against gravity, 4 = against some resistance, Normal = 5.

## Discussion

A total of 14,903 pregnancy outcomes were examined during the study period. The present study revealed a high burden of NTDs in Tigray, Northern Ethiopia. The overall incidence rate of NTDs was found to be 131 per 10,000 births (195/14903). The highest burden of NTDs was observed in Adigrat Hospital from Eastern Zone (174 per 10,000 live and stillbirths) and southern zone of Tigray (304 per 10,000 live and stillbirth). Similar findings were reported from prospective studies of births at three teaching hospitals in Addis Ababa, Ethiopia with 126 per 10,000 births [19]. Folate deficiency is widespread in Ethiopia. The prevalence of severe folate deficiency in Tigray and Ethiopia was reported to be 54% and 46%, respectively [20]. Thus, the high prevalence of folate deficiency could be the reason for the high burden of NTDs in Tigray. The NTDs incidence rate identified in our study is higher than the report from eight World Health Organization member nations in Africa, which revealed an overall prevalence rate of 11.7 per 10, 000 births [1]. The low prevalence rate is shown in most developed and many developing countries may be due to improved health seeking behavior, health and nutrition adequacy and planned pregnancies. In developed countries such as in the United States, China and Canada, it has been reported that after folic acid fortification and supplementation the non folic acid preventable spina bifida and anencephaly rate is 5 per 10,000 births [21]. In contrast, the incidence of a rate of 131 per 10,000 births observed in our study would be an epidemic that is 26 fold higher than the observed in the world. This emphasizes the urgency to implement effective programs to get all women of reproductive age to have adequate folic acid to prevent all of folic acid-preventable spina bifida and anencephaly, which would prevent 96% (125/131) of spina bifida and anencephaly in the Tigray Provence.

Our results showed that males were more susceptible to NTDs compared to their female counterparts. Our finding is consistent with reports from North African and Sub-Saharan countries that showed a predominance of NTDs in males. However, most European reports revealed that females were more affected by NTDs compared to males [22, 23]. The reason given for the female predominance was that the female chromosome creates a risk for epigenetic drag molecular mechanisms of neural fold transformation [24, 25]. This might imply that different mechanisms are involved in the genesis of NTDs.

The findings in this study revealed that NTDs were the main causes of stillbirths and hence infant mortality. Almost a quarter of the infants with NTDs (23%) were live births who suffered from various levels of disability such as distorted neonatal reflexes, and serious limb paralysis or impairment in motor function. This is supported by the report from a comprehensive review of the world medical literature which showed a child with spina bifida present with serious, chronic incapacities, such as limb paralysis, lack of sensation that enhances the risk of pressure sores, hydrocephalus, deformities of the limbs and spinal column, vesicle, intestinal and sexual dysfunctions and learning difficulties with risks of psycho-social maladjustment [26].

The commonest NTD was anencephaly followed by spina bifida in the present study, which is comparable with reports from the Nigerian studies which showed anencephaly as the most common type of neural tube defect [27]. Similarly, The commonest NTD was Spina bifida followed by anencephaly in the retrospective studies from the two teaching hospitals in Addis Ababa, Ethiopia [28]. The reason why anencephaly is predominant as compared to the previous retrospective live birth study in Addis Ababa is that our study included stillbirths which accounted for 77% of all NTDs. Anencephalic newborns are rarely live births. Spina bifida was mostly identified in the lumbar region in our study, which is comparable with the report in India, suggesting that the lumbar region is most prone to spina bifida [29].

The most common associated anomaly observed in our study was hydrocephalus followed by clubfoot, abdominal anomalies (omphalocele), of babies afflicted with spina bifida. This study is comparable with the reports from two teaching hospitals in Addis Ababa, which showed that the spina bifida was associated with hydrocephalus followed by clubfoot [28]. An association between a higher prevalence rate of NTDs and geographical condition, race, sex, certain maternal health, parental socioeconomic condition, parental exposure and poor dietary habits in the Shanxi Province, British islets and USA were reported [30, 31, 32]. Generally speaking, the prevalence of NTDs varies according to the geographic conditions, race, sex, certain maternal conditions, parental socioeconomic status, parental exposure and poor dietary habit [31, 32].

## Limitation of the study

Since this study is facility-based, many babies might have been missed from the survey, especially those medically unattended deliveries, outside of the hospital. This may have resulted in a potential misestimate of the NTD incidence. Numerous important clinical patterns of the NTD was not attempted due to the limited capacity of perinatal surveillance (MRI or other screening, high-level perinatal ultrasound) we have the region. Several demographic and clinical characteristics of the mothers, who have given birth to fetuses with NTD analyzed in this study has not been sought due to the limitation of the resource we faced during the study period.

## Conclusion and recommendation

Assuming that the non folic acid preventable rate should be 5 per 10,000 births, our prevalence rate is 131 per 10,000 births, then we have a rate or an epidemic that is 26 times what it should be. This just emphasizes the urgency to implement effective programs to get all women of reproductive age to have adequate folic acid to prevent all of folic acid-preventable spina bifida and anencephaly, which would prevent 96% (125/130) of spina bifida and anencephaly in the Tigray Provence.
